# Exploration of the factors influencing hearing disability in older adults of China: a nested case-control study

**DOI:** 10.3389/fpubh.2024.1305924

**Published:** 2024-01-17

**Authors:** Wan-Qiong Zhou, Jing Liu, Yi-Tian Gao, Lan-Shu Zhou

**Affiliations:** School of Nursing, Naval Medical University, Shanghai, China

**Keywords:** aging, older adults, disability, hearing, nested case-control

## Abstract

**Objective:**

As two line trends – aging disability and disability aging – continue to emerge, hearing disability is becoming increasingly prevalent among older adults in china. This study aimed to investigate the incidence of hearing disability among older adults and identify the various factors contributing to its development.

**Methods:**

In this matched nested case-control study, data from the China Health and Retirement Longitudinal Study from 2011 to 2018 were analyzed. A total of 4,523 older adults were recruited from a national sample database, of which 1,094 individuals were eligible for inclusion in the hearing disability cohort, while 3,429 older adults who had not been diagnosed with hearing disability were considered non-hearing disability controls. Hearing disability was assessed by a self-reported question. These controls were matched to hearing disability cases in a 1:1 ratio based on age and sex. The logistic regression models were used to find out various factors of hearing disability in the target population.

**Results:**

Totally 1,094 individuals (24.14%) developed hearing disability during the follow-up period. After 1:1 matching, 2,182 subjects were included in the study, with 1,091 cases in the case group. Factors that influenced the incidence of hearing disability in older adults included annual *per capita* household income (OR = 0.985, *p* = 0.003), cognitive function (OR = 0.982, *p* = 0.015), depression level (OR = 1.027, *p* < 0.001), somatic mobility (OR = 0.946, *p* = 0.007), history of kidney disease (OR = 1.659, *p* < 0.001), history of asthma (OR = 1.527, *p* = 0.008), history of accidental injuries (OR = 1.348, *p* = 0.015), whether there is a place for recreational and fitness activities in the community (OR = 0.672, *p* < 0.001), and whether there is a health service center/health center in the community (OR = 0.882, *p* = 0.006).

**Conclusion:**

The incidence of hearing disabilities among older adults in China is high. The protective and risk factors that contribute to the incidence of disability should be fully considered in the care of older adults.

## 1 Introduction

A disabled person is defined as someone who suffers from the loss or abnormality of certain tissues or functions in their psychological, physiological, or bodily structure, resulting in an inability to normally engage in certain activities. In China, persons with disabilities are categorized into six types: hearing disability, visual disability, physical disability, intellectual disability, speech disability and mental disability. Among them, hearing disability is a condition that can result in hearing loss or impairment in both ears due to various reasons, causing difficulty in perceiving sounds from the surrounding environment and speech and hindering normal language communication activities with others (1, 2). In China, hearing loss is defined as a hearing threshold that is outside the normal range, and hearing impairment is defined as a decrease in hearing due to a variety of different causes. Distinguishing between hearing loss and hearing impairment, hearing disability encompasses both.

In the second national sample survey of people with disabilities, there were 20.04 million people with hearing disabilities, accounting for 24.16% of all people with disabilities, the largest number globally (3). More importantly, older adults appear to have become the leading population with hearing disabilities in the country. The incidence of hearing disability increases exponentially with age, with approximately 12.7 percent of older adults aged 60 years and over having a hearing disability globally, increasing to 58 percent at age 90 years (4). With China’s aging population exceeding 200 million and growing at a rate of about 8 million per year, about 30–60% of the older adults have varying degrees of hearing impairment, and 70–80% of the older adults over the age of 75 suffer from hearing loss (5). The first research report on the social problem of hearing loss in the older adult, “Dare to Ask Heavenly Music|Ten Questions on Hearing Health in the older adult,” released at the annual launch of our 2022, shows that about one-third of old adults over the age of 65 years old in China have a hearing disability of moderate or higher, and the number rises to about one-half among old adults over the age of 75 years old (6). Therefore, hearing disability among older adults should be carefully monitored. As disability aging becomes increasingly prevalent in China, hearing disability among older adults is gaining more attention as a major health concern, potentially becoming the third most significant issue after heart disease and arthritis (7, 8). Studies have shown that hearing disability in older adults is not simply a hearing problem; hearing disability can have far-reaching effects on older adults, not only on interpersonal interactions but also on health, independence, well-being, and quality of life (9). Hearing disability is also associated with many psychological disorders, including social isolation, depression, anxiety, and cognitive impairment (10–13).

As aging with disabilities continues to progress, care for the older adult with disabilities is an inescapable issue for society, and hearing disability, as the type of disability that accounts for the largest proportion of older adults among all types of disabilities, its management and future exploration should be focused on. China has always attached great importance to disability prevention. In 2017, China issued the Regulations on Disability Prevention and Rehabilitation of Persons with Disabilities as the first national-level guiding platform for disability prevention, emphasizing the management and supervision responsibilities of governments at all levels in disability prevention and rehabilitation and building a structure covering the whole population and the whole life cycle (14). It is essential to actively prevent and treat hearing disabilities in older adults for better prevention and improve their quality of life. Furthermore, understanding the factors contributing to hearing disability in this population is fundamental to this task. However, the occurrence of hearing disability in the older adult and its influencing factors have been largely underexplored, limiting targeted prevention and effective nursing measures of this population.

To our knowledge, research on hearing disability has been limited to investigations focused on individuals, families, or communities and primarily based on small sample sizes. At the same time, the results of the current study are limited to the physiological level of factors affecting the occurrence of hearing disability in older adults, such as chronic diseases. For example, a survey conducted by Jiang Taogen et al. in Shanghai, China, reported that the factors affecting the incidence of hearing disability in old adults were senile deafness, noise and blast deafness, systemic diseases, otitis media and other factors that affect the development of hearing disability in older adults (15). However, it is now understood that hearing disability results from a combination of multiple factors and levels. The impact of other aspects, such as the social and psychological dimensions, on the occurrence of hearing disability in older adults has been neglected. Due to the limitations of the current study, it cannot be concluded that the findings fully represent the hearing disability situation of the older adult in China. In light of this, the China Health and Retirement Longitudinal Study (CHARLS, 2011–2018) was utilized to observe the occurrence of hearing disability in older adults based on a large population sample and multivariate follow-up data. Our research aims to provide future scholars with a more detailed understanding of the factors associated with the occurrence of hearing disability in older adults and to assist them in developing targeted measures to prevent hearing disability and provide precise care in this population.

## 2 Materials and methods

### 2.1 Study design

This study is a longitudinal retrospective nested case-control study.

### 2.2 Study participants and data source

Data for this study were obtained from the China Health and Retirement Longitudinal Study (CHARLS) database of Peking University in 2018. This survey collects microdata representative of middle-aged and older households and individuals 45 years or older in China. The national baseline survey was conducted in 2011 and covered 150 county-level units and 450 village-level units, with data collected from approximately 10,000 households and 17,000 people. These data are tracked every 2–3 years. We used the baseline data from 2011 and follow-up data from 2013, 2015, and 2018 to establish the observation cohort. We obtained free access to the data from the official CHARLS website^1^ prior to the start of the study.

### 2.3 Study sample

We utilized two questions for screening the study population. Hearing disability was assessed by a self-reported question (Question 1): “Do you suffer from any of the following disabilities? ① Physical disability ② Brain damage/intellectual disability ③ Blind or partially blind ④ Deaf or partially deaf ⑤ Mute or severe stuttering.” People who answered “④” in question 1 were classified as self-reported disabled.

Question 2: “When were you born?” was used to identify older adults. It should be noted that the definition of older adults in this study was in accordance with the Law of the People’s Republic of China on the Protection of the Rights and Interests of the Older Adults (16), which defines “older adults” as citizens who are 60 years of age or older. This legal document establishes 60 years as the age at which older adults are entitled to most social benefits and are encouraged to retire.

We totally obtained 4,523 cases. The case group consisted of any older adult who was defined as having a hearing disability during the three follow-up visits from 2013 to 2018, while those who did not develop a hearing disability until the end of the 2018 follow-up visits were categorized as controls.

**Figure 1:**
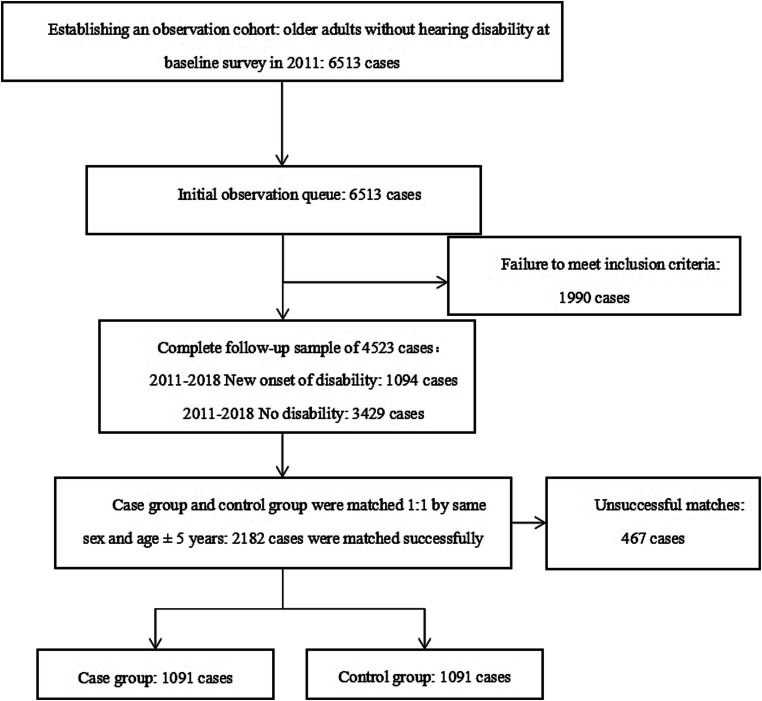
A nested case-control study sample profile of hearing disability.

We employed a 1:1 Propensity Score Matching process by setting up a random seed in the SPSS software based on the principle of highest data utilization to match the case group with the control group according to the same sex and age ± 5 years (caliper = 0.02). The resulting data were then matched. The transformation of the study sample can be seen in Figure 1.

### 2.4 Variables

In this study, the variables were chosen based on the Health Ecology Model, taking into consideration the availability of data from the CHARLS questionnaire. The Health Ecology Model (17) emphasizes that the health of individuals and populations is the result of the interdependence and interaction of individual factors, physical and social environmental factors, and health services, and that these factors are also interdependent and constrained, influencing the health of individuals and populations through multidimensional interactions. He categorized these factors affecting the health of individuals and groups into five layers, starting from the core layer, which includes innate biogenetic factors such as gender, age, height, weight, BMI, and other personal traits. The second layer comprises the behavioral and psychological characteristics of individuals, while the third layer represents the family layer. The fourth layer is related to living and working conditions, and the fifth layer pertains to the socio-economic, cultural, health, environmental, and policy factors at the local, national, and global levels. The research variables and their codes for this study are as follows:

1. Personal traits Personal traits include age, gender, BMI, marital status, education level, and household registration. Age was measured as a continuous variable in the original questionnaire, but we recorded age as a multicategorical variable and divided it into five categories: 1 = 60–65 years old, 2 = 66–70 years old, 3 = 71–75 years old, 4 = 76–80 years old, and 5 = 81 years old and above. Household registration, gender, and marital status are dichotomous variables (household: 1 = agricultural, 2 = non-agricultural; gender: 0 = female, 1 = male; marriage: 0 = no spouse living together, 1 = with spouse living together); BMI and education level are multicategorical variables (BMI was divided into three categories: 1 = low, 2 = normal, 3 = overweight; education level is divided into four categories: 1 = illiterate, 2 = elementary school, 3 = junior high school, 4 = high school and above).
2. Lifestyle and psychological characteristics Lifestyle and psychological characteristics included smoking, drinking, adequate sleep at night, lunch break habit, attending social events, and depression level. Adequate sleep at night and lunch break habit were dichotomous variables (adequate sleep at night: 0 = less than 6 h of sleep, 1 = more than 6 h of sleep; lunch break habit: 0 = no, 1 = yes). Smoking, drinking, and attending social events were multicategorical variables (smoking: 1 = never smoked, 2 = quit, 3 = still smoking; drinking: 1 = yes, 2 = no; attending social events: 1 = none, 2 = 1 kind, 2 = 3 kinds or more). The level of depression was measured as a continuous variable, utilizing the CESD-10 scale developed by Sirodff from the National Institute of Mental Health (18). The scale comprises ten items, and scores range from 0 to 30. Higher scores represent higher levels of depression.
3. Household level Household-level factors include annual *per capita* household income (thousand dollars), number of children, residence, and area of residence. Annual *per capita* household income is a continuous variable and is filled in with actual values. Number of children and residence were assessed as multicategorical variables (1 = no children, 2 = 1, 3 = 2 and more, residence, 1 = living with children, 2 = living in close proximity to children, 3 = living away from children). Area of residence was a dichotomous variable (1 = rural, 2 = urban).
4. Living and working conditions The living and working conditions that were considered in this study included factors such as whether the participant had barrier-free access, access to a toilet or bathing facilities, suffered from comorbidities, other disabilities, history of falls, hip fractures, cataract surgery, glaucoma, accidental injury, as well as measures of cognitive function and somatic mobility. Each factor was analyzed as a dichotomous variable, with scores of 0 representing no and scores of 1 representing yes. Comorbidities included hypertension, dyslipidemia, diabetes mellitus, malignancy tumor, chronic lung disease, liver disease, heart disease, stroke, kidney disease, digestive system disease, emotional/psychiatric disorders, memory-related disease, asthma and arthritis/rheumatism. Other disabilities encompassed visual, speech, intellectual, and physical disabilities. Cognitive function was assessed using the Mini-mental State Examination (MMSE) (18), which has 31 questions to evaluate cognition in areas of temporal orientation, memory, delayed recall, and calculation, with a score range of 0 to 31 and higher scores indicating better cognitive function. Somatic mobility was evaluated using the Short Physical Performance Battery (SPPB) (19), which includes balance, the 4 min walk test, and the sit-to-stand test, with a score range of 0 to 12. Higher scores reflected better somatic function among the older adult.
5. Environmental policy Several factors were assessed as dichotomous variables, such as whether there are organizations that assist the older adult, the sick and the disabled, whether the community or village primarily consists of dirt roads, whether there is a place for recreational and fitness activities in the community, and whether there is a health service center/health center in the community. Each variable was examined as a dichotomous variable, with scores of 0 representing no and scores of 1 representing yes, respectively. Additionally, the study looked at health insurance as a multicategorical variable, with categories including no insurance (scored as 1), urban and rural resident medical insurance (scored as 2), urban workers’ health insurance (scored as 3), and public medical care or commercial insurance (scored as 4).

### 2.5 Statistical analysis

SPSS 22.0 software was used to perform all statistical analyses, and the following statistical methods were used for data analysis. The mean and standard deviation were used to express the measurement data, and the frequency and proportion were used to describe the count data. Mann–Whitney U and Chi-square tests were used for multi-group comparison of categorical variables. A conditional logistic regression analysis of the Cox risk regression model was used to estimate the risk of developing hearing disability in different older adults, and ratio ratios (ORs) and confidence intervals (CIs) were calculated.

In this study, a *p*-value less than 0.05 was statistically significant.

### 2.6 Ethical principles

The ethical application for the CHARLS survey was approved and updated annually by the Institutional Review Board of Peking University.

## 3 Results

### 3.1 Sample characteristics

**Figure 2:**
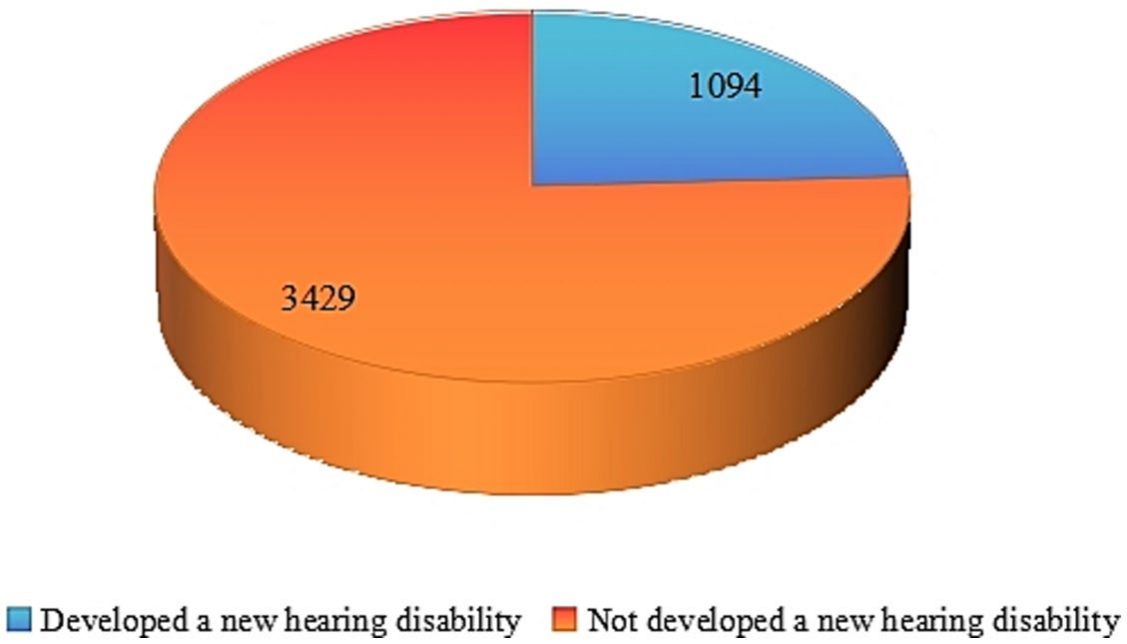
Self-reported incidence of hearing disability among older adults.

Before data matching, the cohort included a total of 4,523 complete samples, of which 1,094 (24.14%) developed a new hearing disability during the follow-up period (Figure 2). After matching, the study included 2,182 subjects, aged between 60 and 96 years, with an average age of 67.28 ± 6.13 years. The study subjects exhibited a slight male predominance (*n* = 552, 50.6%). More details are provided in Table 1.

**Table 1:** Descriptive statistics and univariate analysis of baseline data (*n* = 2,182).

Variables		Case group (*n* = 1,091)		Control group (*n* = 1,091)		Statistics	*p*
		Frequency	Proportion (%)	Frequency	Proportion (%)		
Age (year)	60–65	363	33.3	363	33.3	0 (χ^2^)	1
	66–70	297	27.2	297	27.2		
	71–75	221	20.3	221	20.3		
	76–80	113	10.4	113	10.4		
	81 and above	97	8.9	97	8.9		
Gender	Female	539	49.4	544	49.9	0.046 (χ^2^)	0.830
	Male	552	50.6	547	50.1		
Household registration	Agricultural household	909	83.3	804	73.7	29.944 (χ^2^)	<0.001**
	Non-agricultural household	182	16.7	287	26.3		
Area of residence	Rural	928	85.1	840	77	23.085 (χ^2^)	<0.001**
	Urban	163	14.9	251	23		
Marital status	No spouse	227	20.8	233	21.4	0.099 (χ^2^)	0.753
	With spouse	864	79.2	858	78.6		
Education level	Illiterate	433	39.7	390	35.7	17.690 (χ^2^)	0.001**
	Elementary School	498	45.6	476	43.6		
	Junior High School	118	10.8	144	13.2		
	High School and above	42	3.8	81	7.4		
Health insurance	No insurance	77	7.1	85	7.8	24.707 (χ^2^)	<0.001**
	Urban and rural residents’ medical insurance	896	82.1	817	74.9		
	Urban employee medical insurance	82	7.5	128	11.7		
	Publicly funded medical insurance	19	1.7	45	4.1		
	Commercial insurance	17	1.6	16	1.5		
Annual per capita household income (thousand dollars)		5.03 ± 6.99		7.37 ± 10.57		−4.874 (Z)	<0.001**
Comorbidities	Hypertension	331	30.3	338	31	0.106 (χ^2^)	0.745
	Dyslipidemia	105	9.6	97	8.9	0.349 (χ^2^)	0.555
	Diabetes mellitus	71	6.5	81	7.4	0.707 (χ^2^)	0.400
	Malignant tumor	7	0.6	10	0.9	0.534 (χ^2^)	0.465
	Chronic lung disease	155	14.2	136	12.5	1.431 (χ^2^)	0.232
	Liver disease	50	4.6	40	3.7	1.159 (χ^2^)	0.282
	Heart disease	184	16.9	144	13.2	5.741 (χ^2^)	0.017*
	Stroke	35	3.2	35	3.2	0 (χ^2^)	1
	Kidney disease	100	9.2	60	5.5	10.791 (χ^2^)	0.001**
	Digestive system disease	280	25.7	229	21	6.665 (χ^2^)	0.010*
	Emotional/psychiatric disorders	14	1.3	15	1.4	0.035 (χ^2^)	0.852
	Memory-related diseases	21	1.9	20	1.8	0.025 (χ^2^)	0.875
	Arthritis/rheumatism	449	41.2	391	35.8	6.511(χ^2^)	0.011*
	Asthma	75	6.9	38	3.5	12.777 (χ^2^)	<0.001**
Suffering from other disability	Visual disability	83	7.6	68	6.2	1.601 (χ^2^)	0.206
	Physical disability	50	4.6	39	3.6	1.417 (χ^2^)	0.234
	Intellectual disability	30	2.7	15	1.4	5.105 (χ^2^)	0.024*
	Speech disability	0	0	1	0.1	−1.000 (χ^2^)	0.317
Cognitive function		9.70 ± 5.49		10.87 ± 5.64		−4.827 (Z)	<0.001**
Depression level		10.28 ± 6.87		8.32 ± 6.13		−6.661 (Z)	<0.001**
Somatic mobility		6.15 ± 1.98		6.49 ± 1.97		−3.996 (Z)	<0.001**
BMI	Low	127	11.6	113	10.4	1.891 (χ^2^)	0.388
	Normal	585	53.6	572	52.4		
	Overweight	379	34.7	406	37.2		
History of disability-related illness	History of accidental Injuries (yes)	118	10.8	88	8.1	4.824 (χ^2^)	0.028*
	History of falls (yes)	233	21.4	208	19.1	1.774 (χ^2^)	0.183
	History of hip fracture (yes)	21	1.9	18	1.6	0.235 (χ^2^)	0.628
	History of cataracts (yes)	40	3.7	39	3.6	0.013 (χ^2^)	0.909
	History of glaucoma (yes)	24	2.2	10	0.9	5.856 (χ^2^)	0.016*
Adequate sleep at night (>6 h)	Yes	408	37.4	344	31.5	8.311 (χ^2^)	0.004**
	No	683	62.6	747	68.5		
Lunch break habit	Yes	495	45.4	494	45.3	0.002 (χ^2^)	0.966
	No	596	54.6	597	54.7		
Attend social events	None	590	54.1	531	48.7	6.594(χ^2^)	0.037*
	1 kind	356	32.6	405	37.1		
	2 kinds and more	145	13.3	155	14.2		
Smoke	Never smoked	629	57.7	642	58.8	1.447 (χ^2^)	0.485
	Quit	136	12.5	118	10.8		
	Still smoking	326	29.9	331	30.3		
Drink	Yes	701	64.3	693	63.5	0.593 (χ^2^)	0.743
	No	390	35.7	398	70.1		
Number of children	0	24	2.2	30	2.7	0.892 (χ^2^)	0.640
	1	83	7.6	77	7.1		
	≥2	984	90.2	984	90.2		
Seeing children often	Yes	478	43.8	477	43.7	0.002 (χ^2^)	0.966
	No	613	56.2	614	56.3		
Residence	Living with children	467	42.8	492	45.1	1.167 (χ^2^)	0.558
	Living in close proximity to children	529	48.5	507	46.5		
	Living away from children	95	8.7	92	8.4		
Family environment	Barrier-free access	272	24.9	266	24.4	0.089 (χ^2^)	0.766
	Indoor toilet available	759	69.6	794	72.8	2.736 (χ^2^)	0.098
	With bathing facilities	267	24.5	351	32.2	15.929 (χ^2^)	<0.001**
Community environment	Whether the community or village primarily consists of dirt roads (yes)	289	26.5	223	20.4	11.116 (χ^2^)	0.001**
	Whether there is a place for recreational and fitness activities in the community (yes)	630	57.7	753	69	29.874 (χ^2^)	<0.001**
	Whether there are organizations that assist the older adults, the sick and the disabled (yes)	281	25.8	356	32.6	12.471 (χ^2^)	<0.001**
	Whether there is a health service center/health center in the community (yes)	861	78.9	822	75.3	3.952 (χ^2^)	0.047*

N, number; M, mean; SD, standard deviation; Z, Mann–Whitney U-test; χ^2^, Chi-square test. *Correlation is significant at 0.05 level; **Correlation is significant at 0.01 level.

### 3.2 Description and correlation between the occurrence of hearing disability in older adults and other study variables

Table 1 presents the demographic characteristics that were significantly different between the two study groups, including (1) personal traits level, including education level and household registration; (2) lifestyle and psychological characteristics level, such as adequate sleep at night, attending social events, and depression level; (3) household level, including annual *per capita* household income and area of residence; (4) living and working conditions, specifically whether they have bathing facilities or not; and (5) environmental policy level, which encompasses variables such as the presence of an organization that assists the older adult, sick, and disabled, whether the community/village is primarily composed of dirt roads, the availability of recreational and fitness facilities in the community, and the presence of a health service center/health center in the community.

### 3.3 Indicators of factors influencing hearing disability among the older adults

The conditional logistic regression model was fitted by a Cox risk model. The study used a conditional logistic regression model to analyze the relationship between hearing disability in older adults and 23 independent variables. These variables significantly differed between the case group (those with hearing disability) and the control group (those without) during univariate analysis. More details are described in Table 1.

A stepwise analysis method was applied with an inclusion criterion of 0.05 and an exclusion criterion of 0.10. The results of the conditional logistic regression are presented in Table 2. The findings revealed that annual *per capita* household income (OR = 0.985, 95% CI = 0.975–0.995), cognitive function (OR = 0.982, 95% CI = 0.967–0.996), depression level (OR = 1.027, 95% CI = 1.015–1.039), somatic mobility (OR = 0.946, 95% CI = 0.908–0.985), history of kidney disease (OR = 1.659, 95% CI = 1.265–2.175), history of asthma (OR = 1.527, 95% CI = 1.119–2.084), history of accidental injuries (OR = 1.348, 95% CI = 1.060–1.716), whether there is a place for recreational and fitness activities in the community (OR = 0.672, 95% CI = 0.578–0.782), and whether there is a health service center/health center in the community (OR = 0.882, 95% CI = 1.075–1.528) had a statistically significant effect on the occurrence of hearing disability in older adults.

**Table 2:** Results of conditional logistic regression analysis of factors influencing hearing disability in older adults.

Variables	Regression coefficient (B)	Standard error (SE)	Wald χ^2^	*p*	OR	95% CI
Annual per capita household income (thousand dollars)	−0.015	0.005	9.020	0.003	0.985	0.975–0.995
Cognitive function (scores)	−0.019	0.008	5.863	0.015	0.982	0.967–0.996
Depression level (scores)	0.027	0.006	20.425	<0.001	1.027	1.015–1.039
Somatic mobility (scores)	−0.056	0.021	7.208	0.007	0.946	0.908–0.985
History of kidney disease	0.506	0.138	13.424	<0.001	1.659	1.265–2.175
History of asthma	0.423	0.159	7.119	0.008	1.527	1.119–2.084
History of accidental Injuries	0.299	0.123	5.909	0.015	1.348	1.060–1.716
Whether there is a place for recreational and fitness activities in the community	−0.398	0.077	26.522	<0.001	0.672	0.578–0.782
Whether there is a health service center/health center in the community	0.248	0.090	7.646	0.006	0.882	1.075–1.528

χ^2^ = 166.938, *p* < 0.001.

Of these factors, annual *per capita* household income, cognitive function, somatic mobility, recreational and fitness activities, and health service centers/health centers were protective factors. The risk of hearing disability decreased by 1.5, 1.8, and 5.4% for each one-thousand-dollar increase in annual *per capita* household income, cognitive function, and somatic mobility, respectively. The risk of hearing disability was also reduced by 32.8 and 11.8% for older adults with a place for recreational and fitness activities and health service centers/health centers in the community, respectively.

On the other hand, the risk of hearing disability increased by 2.7% for each unit increase in depression and by 65.9, 52.7, and 34.8% for older adults with kidney disease, asthma, and a history of accidental injury, respectively.

## 4 Discussion

This study was designed to conduct a nested case-control study to analyze the incidence of hearing disability among Chinese older adults and to identify the influencing factors that contribute to this phenomenon, using big data from the CHARLS database. The results showed that the incidence of hearing disability among Chinese older adults was high (24.14%), and the influencing factors included annual *per capita* household income, cognitive function, depression level, somatic mobility, history of kidney disease, history of asthma, history of accidental injuries, whether there is a place for recreational and fitness activities in the community, and whether there is a health service center/health center in the community. China has the largest older adults population in the world and is also one of the fastest-aging countries. In recent years, the Chinese government has prioritized addressing the aging population and enhancing the well-being of the older adult through policy initiatives. However, with the growing prevalence of age-related disabilities, hearing disability has become a significant health concern for older adults. Timely and targeted measures are required to prevent and treat hearing loss in old adults to promote healthy aging. The findings of this study lay the groundwork for developing effective prevention strategies for age-related hearing disability among older adults.

Our results showed that the probability of hearing disability in later life was 24.14%, significantly higher than reported in a study by Jiang et al. (15) in Songjiang, Shanghai, China (19.54%). This may be due to the wide variation in living conditions of older people across China’s cities, with Shanghai being one of the most developed cities in China. Older adults in Shanghai are at a higher economic level and have access to relatively better health care and quality of life, and as a result, the incidence of hearing disability among older adults in Shanghai is lower than the national average. This also side-steps the fact that China should focus on those more underdeveloped cities, actively build public health services, and prioritize issues such as health care in difficult areas. Since the United Nations World Program of Action Concerning Disabled Persons (1983–1992) was introduced in 1983, the primary objective and focus of medicine have been to prevent disabilities and provide prompt treatment (20). Hence, it is imperative to recognize and address hearing disability in older adults in China without delay.

Our study identified factors that influence the occurrence of hearing disability in older adults, which helps us to identify early and provide important interventions for those who may develop hearing disability. Ranked in descending order of importance, these factors were history of kidney disease, history of asthma, history of accidental injuries, whether there is a place for recreational and history of kidney disease, history of asthma, history of accidental injuries, whether there is a place for recreational and fitness activities in the community, whether there is a health service center/health center in the community, somatic mobility, depression level, annual *per capita* household income, and annual *per capita* household income, and cognitive function. Below we describe each of these factors in detail.

In this study, the annual *per capita* household income was a protective factor against hearing disability in older adults, implying that older adults with better household economic conditions were less likely to have hearing disabilities, consistent with the findings of a study by Fukui et al. (21) on hearing disability in Japanese people aged 36 to 84 years. However, a study by He et al. (22) in China on hearing disability in working-age adults showed no association between the incidence of hearing disability and household income, which could be attributed to the fact that the older adults population is often considered “disadvantaged” (23). In contrast with older adults, younger individuals are more resilient to general physical stresses and external trauma (24). However, aging leads to declining functional abilities among older adults (25), resulting in a greater need for medical resources and care services (26). Accordingly, families must provide more significant and better resources to older adults to sustain their well-being and quality of life, including preserving their hearing health, which places a substantial financial burden on households.

Two other protective factors against the onset of hearing disability in older adults are the availability of recreational and fitness activities in the community and somatic mobility. Somatic mobility represents the level of older adults’ ability to be physically active, and the availability of recreational and fitness activities in the community represents the ability of older adults to be able to access resources for sports exercise and participation. These both imply that physical exercise and activity can aid in preventing hearing disability in older adults, consistent with the research by Martinez-Amezcua et al. (27), which revealed that hearing disability is linked to reduced physical function and walking endurance. Additionally, previous studies have indicated that moderate to severe hearing disability in older adults is associated with lower levels of physical activity (28), given that daily activities become increasingly challenging for older adults with poor physical mobility, and physical activity can lead to extra-articular involvement, including the cochlea and cochlear nerve, thereby increasing the risk of developing hearing disability (29). Therefore, to effectively reduce the occurrence of hearing disability, caregivers can develop a rationalized lifestyle exercise program for older adults to improve their overall strength and increase their physical activity.

The present study also revealed that the presence of a health service or health center in the community is also a protective factor against hearing disability in older adults. Firstly, community health facilities, to some extent, reflect the accessibility of health services, and previous research has demonstrated that access to healthcare is strongly linked to positive health outcomes in older adults (30). Besides, the availability of health centers or health homes in the community embodies the ease of access to care for older adults, enabling prompt treatment of hearing disability or other health conditions that affect hearing and reducing the likelihood of developing permanent hearing loss. Therefore, the government and community must prioritize reinforcing primary healthcare facilities to ensure that older adults have timely and comprehensive access to a wide range of healthcare services. At the same time, community workers should strengthen health screening for the older adult so that their hearing is regularly “monitored” to avoid the sudden occurrence of hearing disability.

Our study also substantiated that cognitive function is protective against hearing disability in older adults. In cognitive hearing science, it is believed that the integrity of auditory stimuli depends not only on the precise encoding of signals by functional peripheral systems but also on the decoding of stimuli by the central auditory system, which requires the involvement of higher-level cognitive processes (31). Thus, when cognitive function declines, it negatively affects the coding processes of the central auditory system, leading to an increased risk of hearing disability. Therefore, caregivers should pay attention to enhancing cognitive function in older adults so that hearing disability can be avoided to some extent. According to the recommendations of the WHO Guidelines for Community Integrated Care for Older Adults, communities should conduct regular assessments of the intrinsic abilities of older adults, with a focus on identifying at-risk populations (32). For older adults with cognitive decline, regular cognitive stimulation and cognitive training in orientation, memory, and attention are provided by caregivers and family caregivers working together to slow cognitive decline, which in turn provides a form of hearing protection for older adults.

An increasing body of evidence suggests that a decline in kidney function is linked to the development of hearing disability (33), which may explain why the presence or absence of kidney disease was a risk factor for the development of hearing disability in older adults in the present study. Thodi et al. (34) proposed that this could be due to similarities in the kidney’s physiology, ultrastructure, antigens, and cochlear vascular patterns, making them vulnerable to ototoxicity caused by certain drugs and ultimately leading to hearing disability. Overall, this finding highlights the need for a comprehensive approach to preventing hearing disability in the older adult that takes into account various symptoms and focuses on maintaining overall health, developing good physical examination habits, and conducting regular assessments for early detection and prevention.

The presence of asthma is considered a risk factor for hearing disability in older adults, as previous studies have indicated a link between allergies and sudden hearing disability (35). It is highly conceivable that allergic reactions in asthma patients increase the risk of hearing disability (36), but the extent of this association is still not fully understood and requires further investigation. Nonetheless, the prevention of hearing disability in older adults should take into account the presence of asthma as a potential factor, and caregivers should be attentive when providing care for older adults with asthma. The most important thing to avoid asthma attacks is to stay away from allergens, follow the doctor’s instructions for correct and continuous inhalation medication for long-term control and relief of asthma symptoms, and avoid acute attacks. Nurses are important guides for health management, and can make the older adult realize the importance of adherence to inhaled medication administration through one-on-one explanations and peer education, use video instruction and personal demonstration to make them master the correct method of medication administration, and enhance medication adherence through electronic medication monitors, telephone follow-ups and individualized intervention programs, which is conducive to the control of asthma attacks, and subsequently to avoid causing hearing damage.

Similarly, a higher incidence of hearing disability was observed in older adults with a history of accidental injuries, consistent with previous studies. For instance, blast injuries, mild traumatic brain injuries, and exposure to high-intensity or prolonged noise have been reported to increase the odds of hearing disability (37, 38). Liu et al. (39) revealed that accidental injuries in the older adult are more frequent and have serious consequences, but targeted prevention efforts tailored to different ages, genders, and injury types can significantly reduce their incidence. Therefore, the government and relevant departments should establish and improve the information monitoring system of injuries and risk factors of the older adult as soon as possible, carry out multi-level and multi-faceted interventions for the risk factors causing injuries to older adults, and continuously improve the care and aging mode of older adults, so as to minimize the occurrence of injuries from the root, and then avoid hearing problems caused by unintentional injuries.

Interestingly, our study also found that a higher level of depression was associated with an increased risk of hearing disability in older adults. This is consistent with the findings of Thomas et al. (40). This may be related to the fact that reduced activity, lack of motivation and decreased attention due to depression reduce auditory input and processing in the central auditory pathway, which subsequently leads to impaired auditory function (41). Another possible reason for this is the finding that depression may lead to hippocampal damage through the glucocorticoid cascade, allowing cognitive decline to occur in older adults, and that deterioration in cognitive function directly exacerbates hearing loss (42), which echoes our conclusions above regarding cognitive function. This suggests that nurses should maintain continuous attention to older adult people with depression. Paying attention to the psychology of the older adult and preventing depression in the older adult is an important aspect of family happiness. Nurses can encourage family members to communicate and care more about the older adult, and promote their sense of happiness (43). In addition, given the interrelationship between depression and cognitive functioning, caregivers should focus on both cognitive decline and the onset of depression rather than looking at them separately and individually.

## 5 Implications for public health and future research

This study revealed that the average level of hearing disability among older adults in China varies geographically, and that future exploration of national policies should focus on cities with lower overall strengths in the country, strengthen the popularization of healthcare services nationwide, and improve the average level of health of older adults nationwide. This study proved that the incidence of hearing disability should be the result of a combination of multiple factors, suggesting that the community should pay attention to older adults as a whole, and that the community should actively explore how to improve the supply of community services for older adults, design service programs that meet the physical and mental characteristics of older adults with respect to the factors associated with the incidence of hearing disability, and carry out precise services to prevent the incidence of hearing disability on a point-to-point basis. Meanwhile, given the interrelationship between depression and cognitive function, future research could explore novel programs for bidirectional interventions for depression and cognitive function in older adults, providing a comprehensive intervention approach for the prevention of hearing disability in older adults. In addition, based on the results of this study, and considering reverse thinking, delaying the development of hearing disability or even treating hearing disability by intervening in the factors associated with the development of hearing disability in older adults who have already developed hearing disability seems to be an important topic for the future.

## 6 Limitation

We must acknowledge the limitations of the study. First, our study of disability in older adults was derived from self-reports of older adults. While this allows for a wider sample to be included, there is a degree of discrepancy with the physician’s diagnosis, which may cause some bias. Second, this study was designed to consider only one country, China, and a single population of older adults, and its main findings need to be generalized more and more cautiously to the global and disability-wide population. Third, in order to fully utilize the data in the database, this study was designed to exclude some potentially important variables with significant missing data such as the use of hearing aids, which may limit the comprehensiveness of the findings to some extent. In the end, despite the correlations between the incidence of hearing disability in older adults and various possible factors derived from our longitudinal studies, we were unable to fully explain the underlying biological mechanisms. Therefore, more in-depth experimental studies are needed to confirm these associations.

## 7 Conclusion

The findings of this study on hearing disability in older adults can foster the development of interventions aimed at preventing hearing disability and promoting better health outcomes. We revealed a high incidence of hearing disability among older adults in China, with various protective and risk factors identified. Protective factors included annual *per capita* household income, cognitive function, somatic mobility, availability of recreational and fitness activities, and access to health services in the community. On the other hand, risk factors included kidney disease, asthma, history of accidental injuries, and level of depression. This study provided a clear picture of the current status of disability in older adults and provided a geographical point of reference for future national policy development. The factors associated with the incidence of hearing disability in older adults identified in this study provided a target for prevention and suggested that services should be tailored to address these specific factors. To prevent hearing disability in older adults, it is essential to focus on the factors above and develop targeted measures to reduce the incidence of hearing disability in this population.

## Data availability statement

The original contributions presented in the study are included in the article/supplementary material, further inquiries can be directed to the corresponding author.

## Ethics statement

The studies involving humans were approved by Ethical approval for all the CHARLS waves was granted from the Institutional Review Board at Peking University. The IRB approval number for the main household survey, including anthropometrics, is IRB00001052-11015; the IRB approval number for biomarker collection, was IRB00001052-11014. The studies were conducted in accordance with the local legislation and institutional requirements. Written informed consent for participation was not required from the participants or the participants’ legal guardians/next of kin in accordance with the national legislation and institutional requirements.

## Author contributions

W-QZ: Writing – original draft, Writing – review & editing, Conceptualization, Data curation, Formal analysis, Methodology. JL: Data curation, Methodology, Project administration, Supervision, Writing – review & editing. Y-TG: Data curation, Methodology, Project administration, Supervision, Writing – review & editing. L-SZ: Conceptualization, Data curation, Funding acquisition, Methodology, Project administration, Supervision, Writing – review & editing.

## Footnotes

^1^http://charls.pku.edu.cn/
